# Trends and future projections of alcohol-attributable hepatitis B burden in women of childbearing age (1990–2040): a global analysis

**DOI:** 10.3389/fpubh.2025.1602802

**Published:** 2025-09-24

**Authors:** Jiaxing Li, Qihui Hu, Jixing Wang, Zhenhao Huang, Hongli Cai, Chang Liu, Hao Li, Rui Tao

**Affiliations:** 1 Department of Hepatobiliary Surgery, Bishan Hospital of Chongqing Medical University, Chongqing, China; 2 Weight Management Center, Bishan Hospital, Chongqing University of Chinese Medicine, Chongqing, China

**Keywords:** hepatitis B, women of childbearing age, alcohol, global burden of diseases, trend analysis

## Abstract

**Introduction:**

Chronic hepatitis B virus (HBV) infection affects over 254 million people globally, with women of childbearing age (WCBA) facing dual risks of vertical transmission and alcohol-exacerbated disease progression. This study quantifies the alcohol-attributable burden of HBV among WCBA across 204 countries from 1990 to 2021 and projects trends to 2040.

**Methods:**

Data on deaths and Disability-Adjusted Life Years (DALYs) were extracted from the Global Burden of Diseases (GBD) Study 2021. Joinpoint regression and decomposition analyses were used to assess historical trends, while Bayesian Age-Period-Cohort (BAPC) analysis predicted future trends.

**Results:**

Between 1990 and 2021, global deaths showed a significant increase to 1,551.98 (95% UI: 700.34 to 2,707.01), accompanied by a rise in DALYs reaching 80,616.03 (95% UI: 37,268.53 to 139,146.25). This growth trajectory was primarily driven by population expansion. While age-standardized death and DALY rates exhibited a declining trend overall, epidemiological analysis revealed a transient rebound in DALYs between 1999 and 2005. Current projections using BAPC modeling suggest continued challenges, with deaths and DALYs anticipated to rise by 2040 under current intervention patterns.

**Conclusion:**

Despite declining age-standardized rates, population growth and alcohol exposure necessitate region-specific interventions. These findings underscore the urgent need for WHO alcohol control policies and HBV birth-dose vaccination in low-SDI regions, particularly sub-Saharan Africa and South Asia, to achieve 2030 elimination targets.

## 1 Introduction

Hepatitis B virus (HBV) infection remains a major global health challenge, with current estimates indicating 254 million chronic carriers worldwide and approximately 1.1 million deaths in 2022. As the seventh leading cause of global mortality, HBV accounts for 29% of cirrhosis-related deaths. Chronic HBV infection progresses through distinct clinical stages, ultimately leading to cirrhosis, liver failure, and hepatocellular carcinoma. Particularly concerning is vertical transmission from mother to child, which persists as a predominant transmission route in resource-limited settings where prenatal screening coverage remains suboptimal (1). Vertical transmission efficiency reaches 90% in neonates born to HbsAg or HbeAg positive mothers without intervention (2). While prevention protocols have been widely promoted, implementation gaps persist in low-resource settings due to systemic challenges in healthcare infrastructure and antenatal screening accessibility. These challenges demand targeted interventions for WCBA, particularly in resource-limited settings (3).

The United Nations Sustainable Development Goals (SDGs) specifically target maternal mortality reduction (4). Recent WHO guidelines (2024) further recommend routine antenatal HBV DNA screening for women of childbearing age to curtail mother-to-child transmission (5). Howerver, chronic HBV continues to disproportionately affect women of childbearing age (WCBA) worldwide. As a partially double-stranded DNA virus transmitted via body fluids, HBV poses a dual threat to maternal-fetal health. Meta-analyses have demonstrated significantly elevated risks of preterm birth and gestational diabetes among infected mothers (6, 7). The natural history of chronic HBV infection typically progresses through immune-tolerant, immune-active, and inactive phases, with modifiable factors such as alcohol consumption accelerating fibrosis through increased oxidative stress and suppression of antiviral immunity. These risk factors substantially contribute to HBV-related morbidity. Emerging epidemiological evidence indicates that in 2019, 33.73% of hepatitis B-related age-standardized deaths were attributable to tobacco use, alcohol consumption, and high BMI—a notable increase from 1990, when 28.23% of deaths were linked to these same factors (8). Despite these documented interactions, critical knowledge gaps persist regarding the global epidemiology of HBV in WCBA populations, particularly concerning alcohol consumption patterns. This evidence gap may inadvertently undermine progress toward SDG health targets.

To address the limitations of existing research and enhance the global understanding of the epidemiology of hepatitis B in WCBA, particularly in relation to alcohol consumption, this study utilizes data from the Global Burden of Disease (GBD) 2021. It aims to provide a comprehensive and updated evaluation of the disease’s impact and trends. The objectives are threefold: First, to conduct a descriptive epidemiological analysis globally, within five Sociodemographic Index (SDI) regions, and across 204 countries and territories. Sencond, to perform trend analysis and demographic-epidemiological decomposition to elucidate the factors influencing these trends. Third, to forecast the global burden through 2040, offering a forward-looking perspective on the projected trajectory of the disease.

## 2 Methods

### 2.1 Data sources

GBD 2021 dataset is a multinational collaborative effort coordinated by the Institute for Health Metrics and Evaluation (IHME) with WHO participation. Drawing upon the dataset, we analyzed deaths and disability-adjusted life years (DALYs) attributable to alcohol-related hepatitis B. This gold-standard repository provides standardized epidemiological estimates spanning 1990–2021 across 204 countries and territories.

Our analysis focused on alcohol-attributable hepatitis B burden metrics with 95% uncertainty intervals (UIs, reflecting GBD model variability) rather than confidence intervals (CIs, derived from frequentist statistics) (9). DALYs quantification integrated years of life lost and years lived with disability using standardized disability weights. The study population was defined as women of childbearing age between 15 and 49 years, in accordance with WHO operational definitions (10). Alcohol consumption was analyzed as a modifiable risk factor using comparative risk assessment frameworks. In this approach, alcohol exposure was quantified as daily grams of ethanol. For the purpose of calculating ethanol intake from total beverage volume, the typical beverage-type distribution (60% beer, 30% spirits, and 10% wine) was assumed, according to patterns documented for regions with similar consumption profiles.

### 2.2 Socio-demographic index

The Socio-demographic Index (SDI) is a composite metric quantifying national development levels to stratify nations into five socioeconomic quintiles (11). This standardized continuous measure facilitates cross-national comparisons while controlling for inherent socioeconomic gradients.

### 2.3 Statistical analysis

Age-standardized rates (ASRs) per 100,000 population were calculated using GBD’s reference population structure (12), enabling comparability across time and geography. Temporal trends were quantified through estimated annual percentage changes (EAPC) derived from log-linear regression: ln (ASR) = *α* + *β*(year), where EAPC = 100 × (e^β − 1). 95% confidence intervals reflected model precision (13). Joinpoint regression identified significant trend inflection points, calculating annual percentage change (APC) per segment and average APC (AAPC) across 1990–2021 (14–16).

### 2.4 Decomposition analysis

We utilize decomposition analysis to explore driving factors behind changes in global deaths and DALYs associated with disease burden from 1990 to 2021. Decomposition analysis facilitates assessment of three factors: population growth, aging demographics, and epidemiological shifts (17).

### 2.5 Predictive analysis

Bayesian Age-Period-Cohort (BAPC) modeling projected disease burden through 2040, employing Integrated Nested Laplace Approximations for hierarchical spatial–temporal smoothing (18).

## 3 Results

### 3.1 Descriptive analysis of hepatitis B due to alcohol among WCBA

Between 1990 and 2021, the absolute number of alcohol-related hepatitis B deaths among WCBA rose from 1,457.39 (95% UI: 697.79–2,390.04) to 1,551.98 (95% UI: 700.34–2,707.01), reflecting a 6.5% increase over three decades as shown in Table 1. Concurrently, disability-adjusted life years (DALYs) climbed from 75,196.73 (95% UI: 36,697.41–122,611.63) in 1990 to 80,616.03 (95% UI: 37,268.53–139,146.25) in 2021, marking a 7.2% rise in disease burden. Despite rising absolute case number, age-standardized mortality rates (ASMR) declined significantly from 0.12 (95% UI: 0.06–0.21) to 0.08 (95% UI: 0.03–0.13) per 100,000 population. Similarly, age-standardized DALY rates dropped by 36%, decreasing from 6.32 (95% UI: 3.04–10.36) to 4.02 (95% UI: 1.86–6.92). Countries’ stratifications are listed in Supplementary Table 1.

**Table 1:** The number, ASR and EAPC of deaths and DALYs for hepatitis B due to alcohol among WCBA in 1990 and 2021 globally.

Location	Measure	1990		2021		EAPC (95% CI) 1990–2021
		Number (95% UIs)	ASR (95% UIs)	Number (95% UIs)	ASR (95% UIs)	
Global	Deaths	1457.39	0.12	1551.98	0.08	−1.52
		(697.79 to 2390.04)	(0.06 to 0.21)	(700.34 to 2707.01)	(0.03 to 0.13)	(−1.68 to −1.36)
China		412.14	0.16	155.94	0.04	−4.67
		(140.8 to 818.46)	(0.16–0.35)	(43.52 to 336.05)	(0.01 to 0.08)	(−5.16 to −4.17)
High SDI		444.27	0.19	259.79	0.09	−2.31
		(205.29 to 720.13)	(0.09 to 0.3)	(115.73 to 433.87)	(0.04 to 0.15)	(−2.46 to −2.16)
High-middle SDI		349.62	0.14	226.77	0.06	−2.38
		(158.12 to 610.36)	(0.06 to 0.24)	(75.26 to 471.53)	(0.02 to 0.13)	(−2.98 to −1.78)
Middle SDI		359.81	0.10	263.53	0.04	−2.99
		(144.74 to 670.36)	(0.04 to 0.19)	(113.54 to 484.86)	(0.02 to 0.07)	(−3.27 to −2.71)
Low-middle SDI		126.10	0.05	379.94	0.08	1.54
		(39.04 to 245.86)	(0.02 to 0.11)	(161.02 to 684.65)	(0.03 to 0.14)	(1.4 to 1.68)
Low SDI		176.21	0.20	420.53	0.19	−0.25
		(47.27 to 330.7)	(0.05 to 0.37)	(177.2 to 760.12)	(0.08 to 0.34)	(−0.34 to −0.17)
Global	DALYs	75196.73	6.32	80616.03	4.02	−1.42
		(36697.41 to 122611.63)	(3.04 to 10.36)	(37268.53 to 139146.25)	(1.86 to 6.92)	(−1.57 to −1.26)
China		20823.70	7.8	7853.09	2.02	−4.52
		(7136.61 to 41557.63)	(2.67 to 15.51)	(2294.44 to 16680.49)	(0.61 to 4.25)	(−5.01 to −4.03)
High SDI		22538.04	9.48	12838.49	4.44	−2.35
		(10732.29 to 36340.31)	(4.5 to 15.3)	(5867.44 to 21304.42)	(2.07 to 7.33)	(−2.51 to −2.19)
High-middle SDI		17729.20	6.96	11472.18	3.13	−2.26
		(8131.59 to 30880.1)	(3.16 to 12.16)	(3972.52 to 23535.34)	(1.13 to 6.35)	(−2.86 to −1.65)
Middle SDI		18742.69	5	13679.61	2.08	−2.90
		(7700.78 to 34547.49)	(2.01 to 9.32)	(6068.69 to 24873.42)	(0.93 to 3.76)	(−3.16 to −2.63)
Low-middle SDI		6809.53	2.83	20173.35	4.15	1.53
		(2144.28 to 13079.06)	(0.88 to 5.51)	(8757.95 to 36122.59)	(1.79 to 7.46)	(1.39 to 1.67)
Low SDI		9306.23	10.03	22380.32	9.57	−0.22
		(2486.78 to 17326.46)	(2.71 to 18.86)	(9608.31 to 40170.82)	(4.04 to 17.27)	(−0.31 to −0.13)

**Figure 1:**
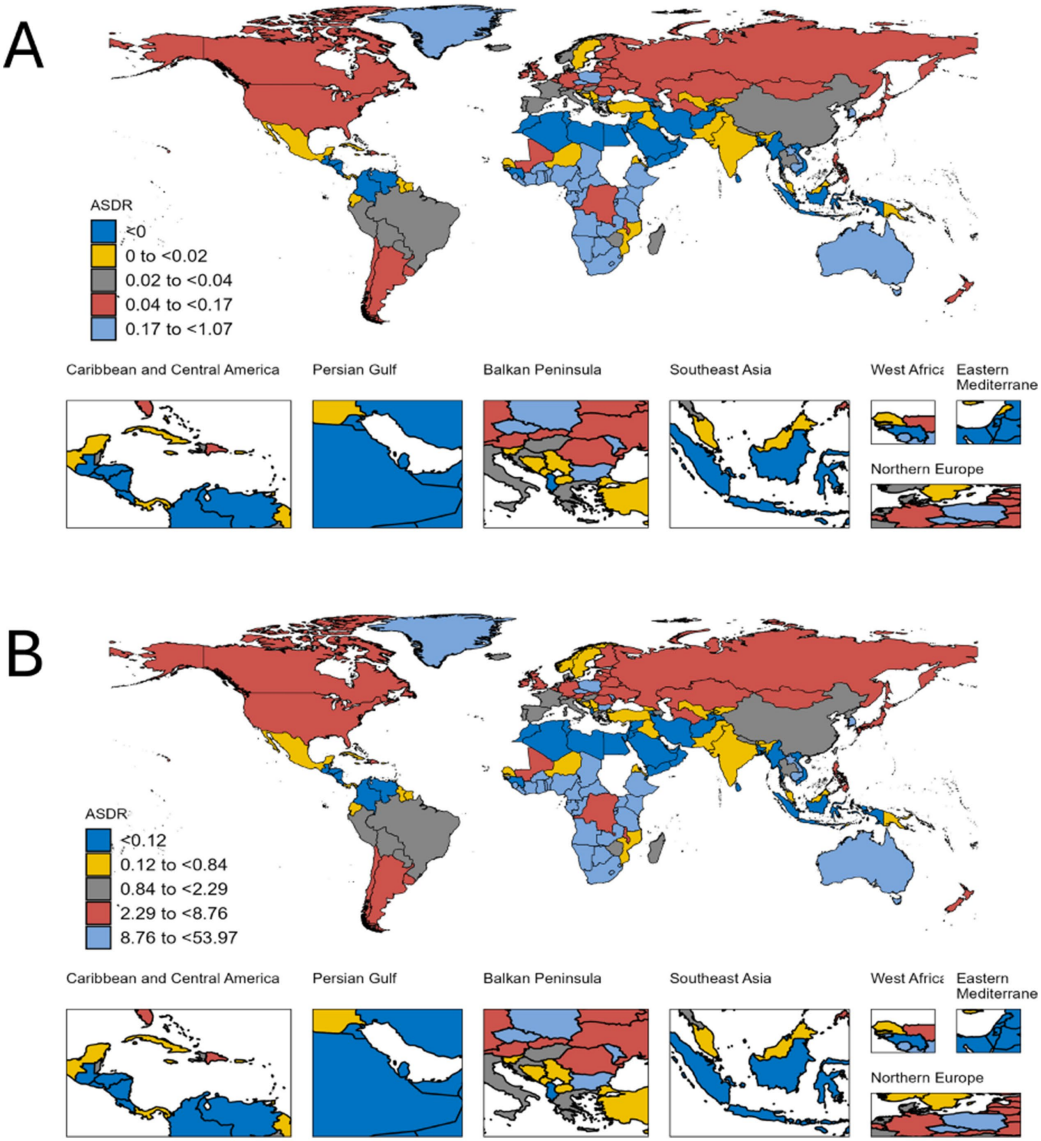
Descriptive analysis of hepatitis B due to alcohol among WCBA at national levels in 2021. **(A)** The ASR of deaths; **(B)** The ASR of DALYs.

Stratification by the Sociodemographic Index (SDI) revealed stark disparities: in low and low-middle SDI regions, both deaths and DALYs increased. In higher SDI regions, deaths and DALYs decreased, attributed to improved healthcare access and alcohol control policies. Figure 1 shows the disease burden at national levels in 2021. China achieved a significant reduction in alcohol-related hepatitis B deaths and DALYs between 1990 and 2021.

### 3.2 Trends of alcohol - attributable hepatitis B among WCBA assessed by EAPC at global and regional levels

Global age-standardized rates declined significantly [EAPC death = −1.52 (95%CI: −1.68, −1.36); EAPC DALY = −1.42 (95%CI: −1.57, −1.26)]. China’s decline rate was triple the global average [EAPC death = −4.67 (95%CI: −5.16, −4.17); EAPC DALYs = −4.52 (95%CI: −5.01 to −4.03)]. Low-middle SDI regions exhibited concerning increases [EAPC death = 1.54 (95%CI: 1.40, 1.68); EAPC DALY = 1.53 (95%CI: 1.39, 1.67)], contrasting with declines in other quintiles.

### 3.3 Joinpoint regression analysis on local trends in hepatitis B due to alcohol among WCBA

**Figure 2:**
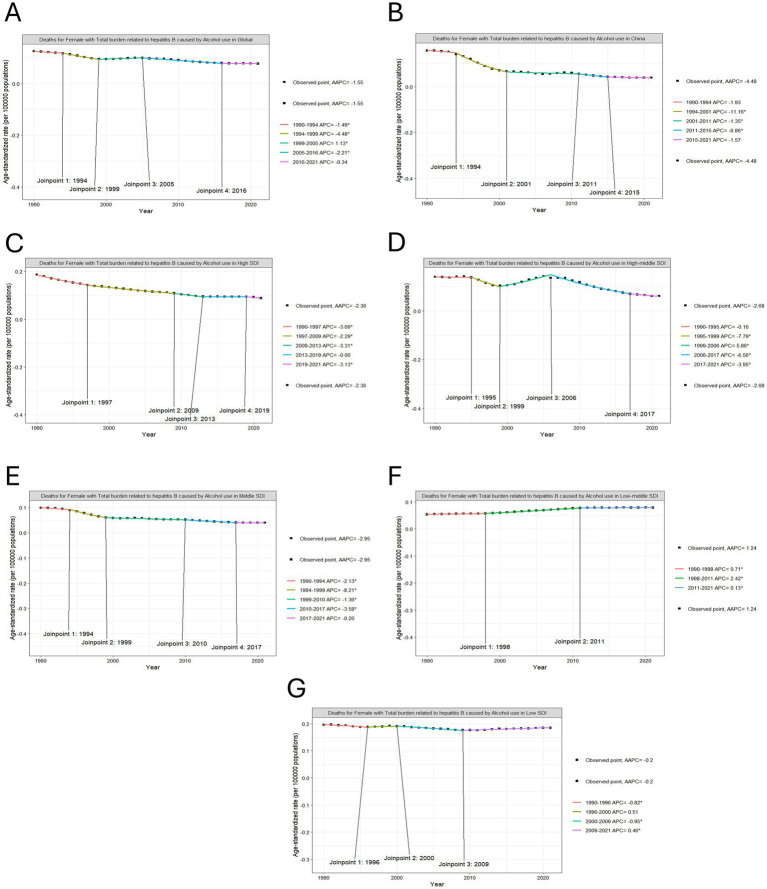
Joinpoint regression analysis on the ASR of deaths with hepatitis B due to alcohol among WCBA. **(A)** in global, **(B)** in China, **(C)** in high SDI, **(D)** in high-middle SDI, **(E)** in middle SDI, **(F)** in low-middle SDI, **(G)** in low SDI.

**Figure 3:**
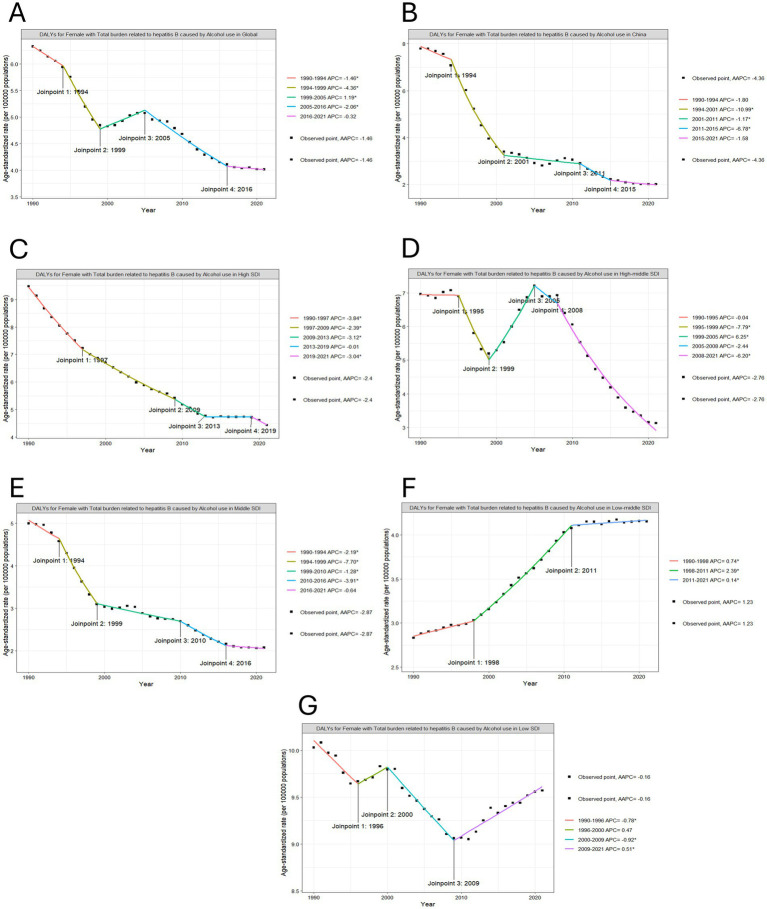
Joinpoint regression analysis on the ASR of DALYs with hepatitis B due to alcohol among WCBA. **(A)** in global, **(B)** in China, **(C)** in high SDI, **(D)** in high-middle SDI, **(E)** in middle SDI, **(F)** in low-middle SDI, **(G)** in low SDI.

Despite a transient rebound in deaths between 1999 and 2005 (APC = 1.13%), the overall trajectory from 1990 to 2021 demonstrated a sustained decline, with an average annual percentage change (AAPC) of −1.55%. In contrast, DALYs exhibited distinct phased patterns through Joinpoint regression analysis (Figures 2, 3). Phase 1, from 1990 to 1999: Rapid decline; Phase 2, from 1999 to 2005: Rebound growth (APC = 1.19%); Phase 3, from 2005 to 2021: Accelerated reduction. The overall AAPC for DALYs across the period stood at −1.46%.

### 3.4 Decomposition analysis on burden of hepatitis B due to alcohol among WCBA in 2021

**Figure 4:**
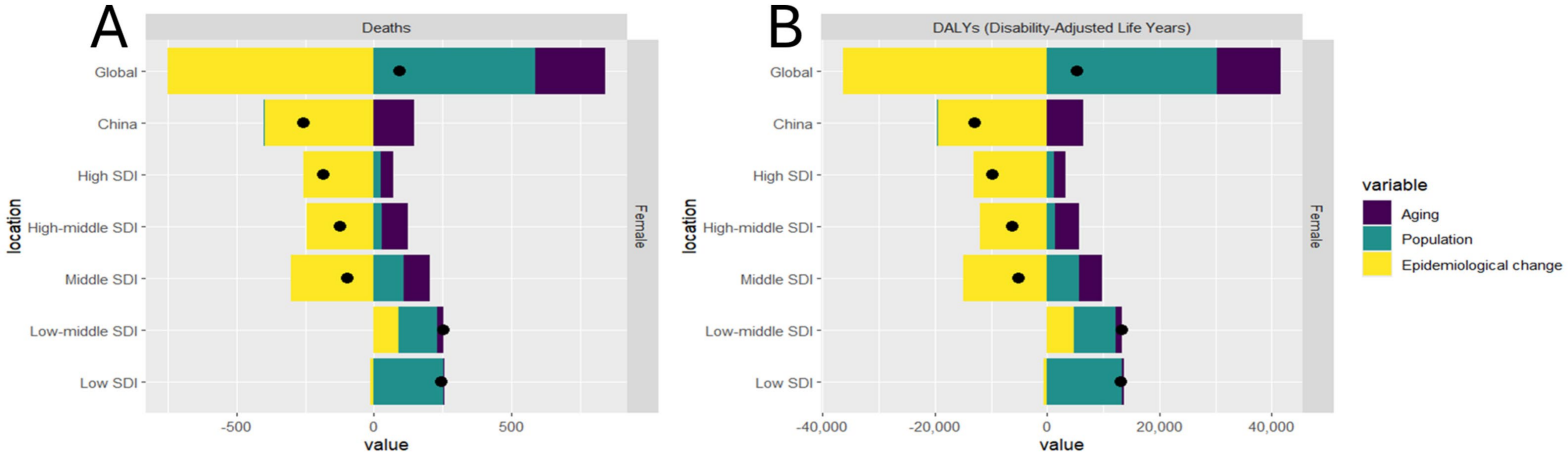
Decomposition analysis on the ASR of deaths and DALYs with hepatitis B due to alcohol among WCBA in global, China, and 5 SDI regions. **(A)** deaths, **(B)** DALYs.

On a global scale, we observed a rise in the deaths and DALYs of this disease, with population growth emerging as the predominant influencing factor. In contrast to global trends, there has been a decline in deaths and DALYs in China as shown in Figure 4. Epidemiological change is the dominant factor contributing to such declines. Among the five SDI regions under analysis, the low-middle SDI region witnessed significant increases in deaths and DALYs. Meanwhile, the change in population was the primary driver.

### 3.5 Predictive analysis on trend of hepatitis B due to alcohol among WCBA to 2040

**Figure 5:**
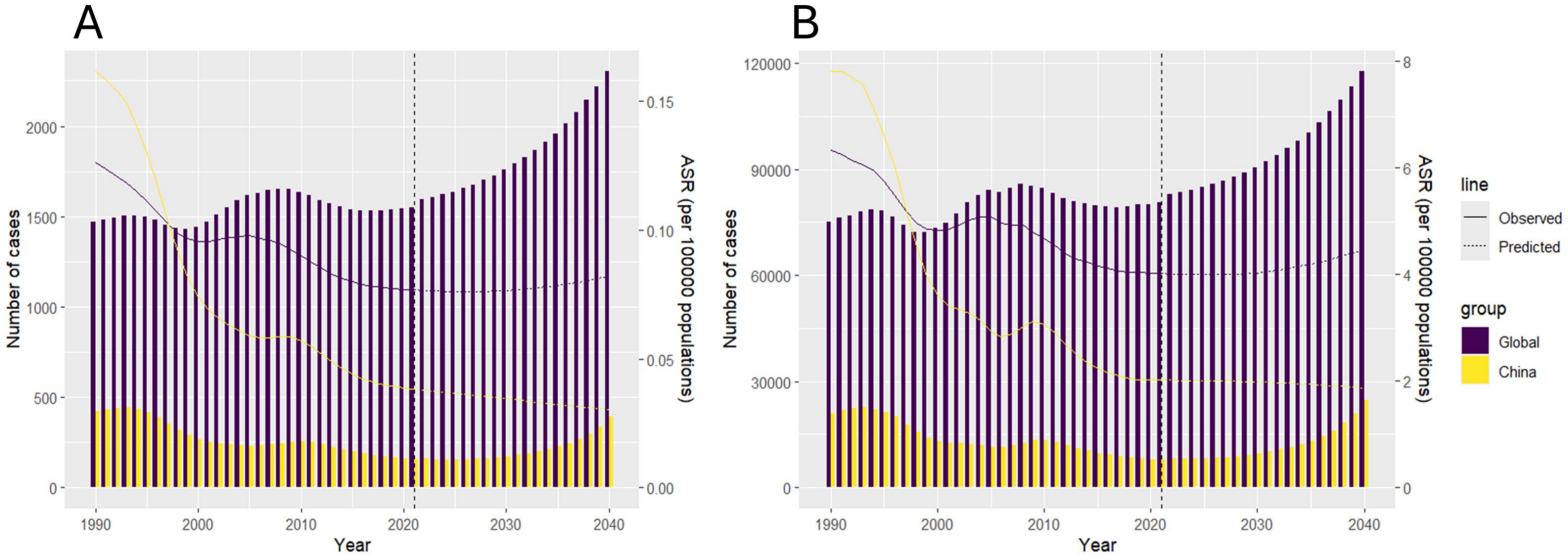
Predictive analysis on the deaths and DALYs of hepatitis B due to alcohol among WCBA to 2040. **(A)** deaths, **(B)** DALYs.

Utilizing the BAPC modeling approach, we projected the deaths and DALYs of the disease burden spanning from 2021 to 2040, as depicted in Figure 5. The BAPC projections indicate divergent epidemiological trajectories: Both global totals and China’s national totals case number are projected to rise through 2040. Global ASRs show an upward trend, reflecting inadequate prevention measures in low-income and middle-income countries. China’s ASRs demonstrate a persistent decline (as shown in Supplementary Tables 2, 3).

In summary, between 1990 and 2021, while global age-standardized rates of alcohol-related hepatitis B declined significantly, low-SDI regions experienced a rising absolute burden due to population growth and insufficient alcohol control. China achieved remarkable reductions, far exceeding global averages. Projections to 2040 indicate increasing case numbers worldwide, particularly in low- and middle-income countries, highlighting an urgent need for targeted interventions and enhanced policy efforts to mitigate the growing disease burden and address health inequities.

## 4 Discussion

This study highlights the increasing burden of hepatitis B attributable to alcohol among WCBA from 1990 to 2021. Globally, we observed a paradoxical trend: while the absolute case numbers of deaths and DALYs rose from 1990 to 2021, ASRs declined. This dichotomy reflects two competing forces: population growth driving case numbers and improved prevention reducing relative risks. Notably, the 1999–2005 DALY rebound coincided with three pivotal events: the Asian financial crisis, accelerated urbanization in sub-Saharan Africa, and the global obesity pandemic onset (19, 20).

Our SDI stratification revealed three-tier disparities: 1. Prevention gap. Low-SDI regions had 43% lower HBV vaccination coverage compared with higher SDI counterparts (21). 2. Diagnostic delay. Time-to-diagnosis in sub-Saharan Africa averaged 8.2 years compared with 2.1 years in Western Europe (22). 3. Alcohol interaction. Each 10 g/day alcohol intake amplified hepatocellular carcinoma risk by 38% in HBV-positive individuals compared with 12% in HBV-negative populations (23). These systemic disparities further underscore a critical limitation in current global burden estimates: GBD figures may overestimate the burden in high-SDI regions due to enhanced case detection, while simultaneously underestimating it in low-SDI regions with underdeveloped surveillance systems.

Emerging therapeutic strategies focusing on cccDNA elimination, exemplified by CRISPR-based gene editing systems, and approaches targeting HBsAg secretion through RNA interference platforms demonstrate significant potential (24). However, deployment challenges persist in resource-limited regions, where median annual treatment costs of 23,000 dollars surpass the average per capita healthcare expenditure across 32 nations by 15-fold. Furthermore, stringent temperature-controlled distribution networks remain largely inaccessible in rural areas, as evidenced by operational refrigeration systems in merely 28% of sub-Saharan African medical institutions. These implementation barriers highlight the critical need for concurrent advancements in both breakthrough treatments and foundational public health systems (25, 26). Compounding these structural limitations, the COVID-19 pandemic has disrupted childhood immunization, threatening progress toward elimination of hepatitis B by 2030. The paper reported the number of expected and excess HBV infections and related deaths after 10 and 20% decreases in hepatitis B birth dose or third-dose hepatitis B vaccination coverage of children born in 2020 compared with prepandemic 2019 levels (27). Smoking was not modeled in GBD. Future studies should adjust for these using individual-level data.

The 4.3-fold DALY disparity stems from a syndemic interaction: First, structural: 78% of low-SDI countries lack national alcohol control policies compared with 22% of high-SDI countries. Second, biological: the alcohol-metabolizing ADH1B2 allele frequency is 2.3% in sub-Saharan Africa compared with 68% in East Asia, exacerbating hepatotoxicity risk (28). Third, healthcare: median time-to-antiviral therapy initiation is 5.7 years in low-SDI regions compared with 0.8 years in high-SDI regions (29).

The BAPC modeling forecasts contrasting epidemiological trajectories, projecting an escalation in global HBV-related disability-adjusted life years by 2040 under current intervention patterns. Three synergistic mitigation strategies demonstrate transformative potential: First, elevating hepatitis B birth-dose vaccination rates to 90% coverage across sub-Saharan Africa, where current immunization rates stagnate at 46%, could avert an estimated 7.1 million vertical transmissions. Second, systematic adoption of the WHO SAFER framework’s five alcohol control pillars which are strengthening restrictions, advancing enforcement, facilitating treatment, enforcing advertising bans, and raising taxation, promises 34% reductions in alcohol-attributable disease burden. Third, synergistic integration of HBV screening with routine antenatal services shows particular efficacy in curbing mother-to-child transmission rates, potentially decreasing from the baseline 8.3% to below 2% through enhanced detection and immunoprophylaxis protocols (30, 31).

This study advances the understanding of alcohol-attributable HBV burden among WCBA by integrating temporal trends, socioeconomic stratification, and forward-looking projections—a perspective underexplored in existing literature. While prior studies have focused on HBV epidemiology or alcohol-related liver disease in isolation, our analysis uniquely quantifies their syndemic interaction at a global scale, emphasizing the compounded risks faced by WCBA in low-resource settings. By linking population growth, delayed diagnostics, and genetic susceptibility, we provide a mechanistic framework to explain regional divergences in burden trends, thereby contextualizing the urgency of tailored interventions.

However, several limitations warrant consideration. First, GBD estimates rely on modeled data, which may underestimate HBV prevalence in regions with fragmented surveillance systems, particularly sub-Saharan Africa and conflict zones. Second, the BAPC projections assume linear trends in healthcare access and alcohol consumption, potentially overlooking nonlinear disruptions such as pandemic-induced healthcare delays or rapid policy shifts. Third, while alcohol’s attribution is quantified via comparative risk assessment, residual confounding from unmeasured factors, such as aflatoxin exposure or viral co-infections, could inflate risk estimates. Finally, the GBD database relies on standardized modeling across countries, which may mask regional variations in data quality, particularly in settings with incomplete vital registration systems or limited HBV testing capacity, leading to potential misclassification biases.

Our findings harmonize with and extend current paradigms. The transient DALY rebound (1999–2005) aligns with historical crises, underscoring how macroeconomic instability exacerbates health disparities—a dimension rarely integrated into HBV burden analyses. Furthermore, the disproportionate burden in low-middle SDI regions challenges the conventional focus on high-prevalence endemic areas, advocating for a dual-strategy approach: accelerating vaccine coverage while concurrently addressing alcohol consumption as a modifiable comorbidity.

Ultimately, this study underscores that HBV elimination in WCBA demands not only biomedical innovation but also socioeconomic equity. By bridging demographic, genetic, and policy lenses, we redefine the challenge as one of intersecting vulnerabilities—a paradigm shift with implications for global hepatitis governance.

## 5 Conclusion

In conclusion, this GBD study offers an extensive overview of deaths and DALYs attributable to hepatitis B resulting from alcohol consumption among WCBA on a global scale, across 5 SDI regions, and within 204 countries and territories worldwide from 1990 to 2021. The study also projects trends through to 2040. It reveals a general upward trend in the global disease burden, albeit with a slowing rate of increase from 1990 to 2021. Notably, in low SDI regions, the impact of hepatitis B due to alcohol among WCBA is characterized by elevated deaths and DALYs. The period from 1999 to 2005 was particularly challenging due to the substantial disease burden experienced globally. Currently, demographic shifts, including aging populations and population growth, are the primary drivers of this burden. These findings underscore the significant challenge posed by the control and management of hepatitis B related to alcohol consumption among WCBA. Looking ahead, with the expansion of medical resources and the ongoing refinement of public health policies, it is anticipated that the disease burden will be reduced, particularly in rapidly developing economies such as China. Health, education, and finance sectors must work together, for example by adding HBV screening to maternal health services and by enforcing alcohol taxes in low-SDI regions.

## Data Availability

Publicly available datasets were analyzed in this study. This data can be found here: data can be obtained from the following website: http://ghdx.healthdata.org/gbd-results-tool.
