# Correction: Trends and future projections of alcohol-attributable hepatitis B burden in women of childbearing age (1990–2040): a global analysis

**DOI:** 10.3389/fpubh.2025.1730460

**Published:** 2025-11-06

**Authors:** Jiaxing Li, Qihui Hu, Jixing Wang, Zhenhao Huang, Hongli Cai, Chang Liu, Hao Li, Rui Tao

**Affiliations:** 1 Department of Hepatobiliary Surgery, Bishan Hospital of Chongqing Medical University, Chongqing, China; 2 Weight Management Center, Bishan Hospital, Chongqing University of Chinese Medicine, Chongqing, China

**Keywords:** hepatitis B, women of childbearing age, alcohol, global burden of diseases, trend analysis

In the published article, there was a mistake in the **Affiliations**. The affiliation of Rui Tao was displayed as “Rui Tao^2^”. The correct statement is “Rui Tao^1, 2^”.

The original version of this article has been updated.

